# Effect of septal flash on right ventricular systolic function in left bundle-branch block patients with preserved left ventricular ejection fraction

**DOI:** 10.1038/s41598-017-06362-1

**Published:** 2017-07-19

**Authors:** Guang-yuan Li, Yong-huai Wang, Zheng-yu Guan, Xuan-yi Jin, Yang Li, Shuang Liu, Chun-yan Ma, Jun Yang

**Affiliations:** Department of Cardiovascular Ultrasound, The First Hospital of China Medical University, Shenyang, Liaoning People’s Republic of China

## Abstract

A leftward motion of the ventricular septum prior to ejection, known as the septal flash (SF), is frequently observed in patients with left bundle-branch block (LBBB). We investigated whether the abnormal motion of the ventricular septum affects right ventricle (RV) contractile performance in LBBB patients with preserved left ventricular ejection fraction (LVEF). Forty-four patients with complete LBBB were selected using standard 12-lead electrocardiograms (ECGs), with 30 healthy individuals serving as controls. According to the presence of SF, patients with LBBB were allocated to two subgroups: those with SF (LBBB-SF, n = 24) and those without SF (LBBB-NSF, n = 20). RV longitudinal strain (LS) decreased in LBBB patients with preserved LVEF compared to control subjects (p = 0.002). And RV LS decreased significantly in LBBB-SF patients compared to NSF-LBBB patients (p = 0.04). RV LS correlated negatively with involved septal myocardial segments of SF (r = −0.36, p = 0.02), but did not correlate with the magnitude of SF. RV contractile performance deceased in LBBB patients with preserved LVEF. SF, particularly the extent of this phenomenon, may further affect RV contractile performance.

## Introduction

Left bundle-branch block (LBBB) is a conductive disorder that causes electric activation dyssynchrony between the right ventricle (RV) and the left ventricle (LV), and between the septum and the left ventricle free wall^1–4^. Such abnormal electric conduct and activation asynchrony may lead to early active leftward contraction of the septum within the isovolumic contraction time, followed by lengthening, while the late-activated lateral wall starts to contract^5^. Abnormal motion of the ventricular septum has been described by echocardiography and is referred to as a septal flash (SF)^6^. A previous study has reported that SF may increase LV inefficient pump function, and may be a pathophysiological mechanism of underlying heart failure in patients with LBBB^5, 7–9^. However, whether SF simultaneously affects RV contractile performance in LBBB patients with preserved left ventricular ejection fraction (LVEF) has not been elucidated.

It has been revealed that two-dimensional speckle-tracking echocardiography (2D STE) can assess RV myocardial global and regional systolic function by obtaining the myocardial strain and strain rate based on the tracking of speckles in grayscale 2D echocardiographic images, and RV longitudinal strain (LS) is less confounded by overall heart motion^10^.

Given these recent findings, using 2D STE and then measuring RV LS, we conducted this study with three objectives in mind: (1) to assess RV contractile performance in LBBB patients with preserved LVEF; (2) to further investigate the effect of SF on RV contractile performance; (3) to investigate the correlation of RV contractile performance with the extent and magnitude of SF. To the best of our knowledge, this is the first study to explore this topic.

## Materials and Methods

### Study Population

Patients with a diagnosis of complete LBBB, and whose LVEFs were within the normal range, were consecutively selected from the outpatient and inpatient departments of The First Hospital of China Medical University. Complete LBBB was defined by baseline standard supine 12-lead electrocardiograms (ECG) as (1) QRS duration ≥140 ms in men or ≥130 ms in women; (2) QS or rS in leads V1 and V2; and (3) mid-QRS complex slurring or notching in ≥2 of contiguous leads V1, V2, V5, V6, I, and aVL^11^. A normal LVEF was defined according to the recommendations of the American Society of Echocardiography criteria: LVEF ≥52% in men or ≥54% in women^10^. Exclusion criteria were as follows: myocardial infarction, positive exercise test result, pulmonary hypertension, acute coronary syndrome, valvular dysfunction, uncontrolled hypertension, ventricular preexcitation, atrioventricular conduction abnormalities, atrial fibrillation, paced rhythm, idiopathic cardiomyopathy, congenital heart disease, poor echocardiographic imaging or ECG, and patient unwillingness to provide informed consent. Several healthy individuals were selected for the control group, with the same exclusion criteria. Ultimately, a total of 50 patients with complete LBBB and normal LVEF (30 females, 20 males; mean age 58 ± 11 years) and 33 age- and sex-matched healthy controls (18 females, 15 males; mean age 58 ± 8 years) were enrolled. All methods were carried out in accordance with the relevant guidelines and regulations. Written informed consent was obtained from all participants, and the study was approved by the China Medical University Ethics Committee.

### Echocardiography

All standard echocardiographic images were acquired in the left decubitus position during normal respiration using a Vivid 7 Dimension ultrasound system (GE Healthcare, Waukesha, WI, USA) equipped with a 2- to 4-MHz phased-array probe, in accordance with the recommendations of the American Society of Echocardiography^10^. At least three consecutive cardiac cycles were stored in cineloop format for offline analysis.

LV end-diastolic volume, LV end-systolic volume, and LVEF were computed from apical two- and four-chamber views using the biplane modified Simpson method. RV basal cavity diameter (RVD1), RV mid cavity diameter (RVD2), RV end-diastolic area (RVEDA), and RV end-systolic area (RVESA) were measured in the RV-focused apical four-chamber view. RV fractional area change (RVFAC) and the index of global RV systolic function were calculated as follows: RVFAC (%) = (RVEDA − RVESA)/RVEDA. Tricuspid annular plane systolic excursion (TAPSE), the index of RV longitudinal systolic function, was measured by M-mode echocardiography using the apical approach with the cursor optimally aligned along the direction of the tricuspid lateral annulus.

Pulsed-wave TDI images were acquired by activating the TDI functions of the echocardiography unit. For the apical approach, the sample volume should be positioned on the tricuspid lateral annulus to record peak systolic velocity of the tricuspid annulus (S’), isovolumic contraction time (ICT), isovolumic relaxation time (IRT), and ejection time (ET) intervals, to achieve a view that shows parallel alignment of Doppler beam with RV free wall longitudinal excursion. RV index of myocardial performance (RIMP), the index of global RV performance, is calculated as follows: RIMP = (IVRT + IVCT)/ET.

### Two-Dimensional Speckle-Tracking Echocardiography

**Figure 1:**
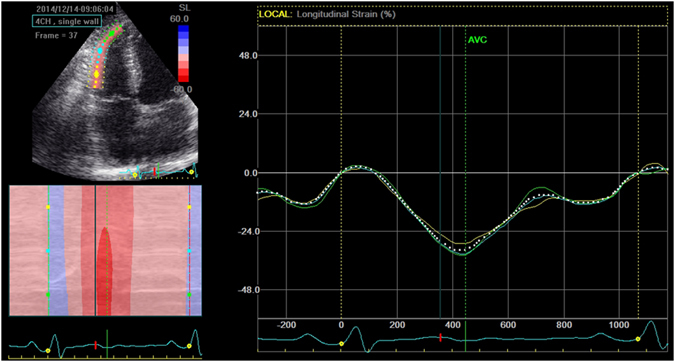
RV longitudinal strain curve by 2D STE. RV, right ventricle; 2D STE, two-dimensional speckle-tracking echocardiography.

Dynamic 2D ultrasound images of three cardiac cycles from the RV-focused apical four-chamber view were acquired using conventional ultrasound, with a frame rate of 57 to 72 frames per second. Image analysis was performed off-line using customized software within the EchoPAC work station (GE Healthcare). The endocardial boundary of the RV was delineated manually, after which the software automatically drew the epicardial boundary. The widths of the regions of interest were adjusted manually to match the actual endocardial and epicardial boundaries. Automatic frame-by-frame tracking of speckle patterns during the cardiac cycle yielded a measure of strain. Subjects with inadequate tracking of more than one segment in the RV-focused apical four-chamber view were excluded from the study. The peak systolic LS of the basal, middle, and apical portions of the RV lateral wall were obtained (Fig. 1). By averaging these segmental values, RV LS was calculated. Similar to STE-derived RV analysis, we obtained strain curve of the 18 segments of LV and measured peak systolic LS. And LV global LS was calculated by averaging all LV segmental values in all views (including apical four-chamber, two-chamber, and long-axis views).

### Assessment of septal flash

**Figure 2:**
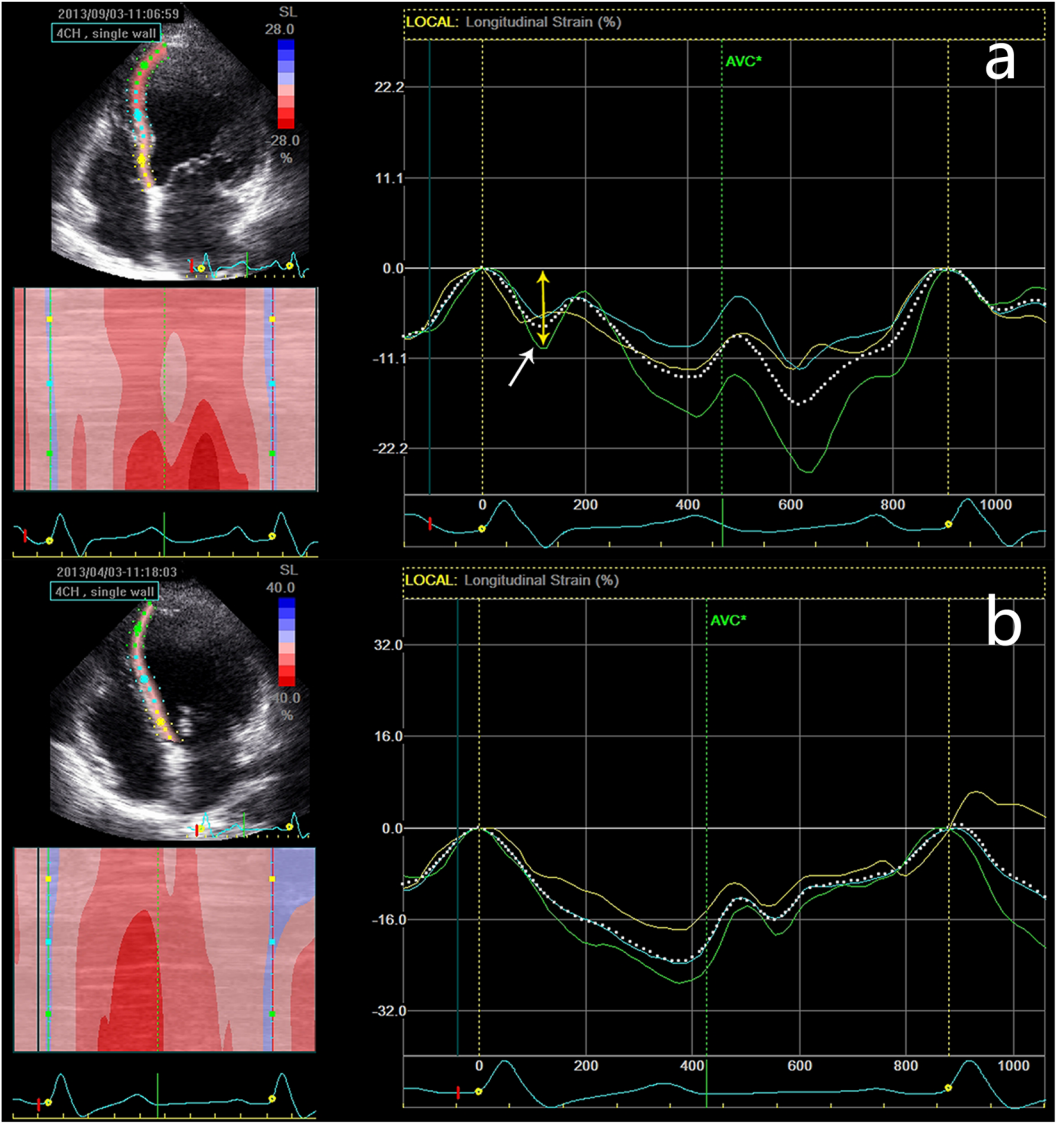
SF by 2D STE. Note the septal strain curve of LBBB-SF (**a**) and LBBB-NSF (**b**) patients. SF was defined as the presence of early leftward and then rightward septal motion within the isovolumic contractile period (white arrow). The magnitude of SF was defined as the maximal early negative peak strain in the involved septal myocardial segments (yellow arrow). LBBB, left bundle branch block; SF, septal flash; 2D STE, two-dimensional speckle-tracking echocardiography.

The existence of SF was assessed by 2D STE in the apical four- and three-chamber views. SF was defined as the presence of early leftward motion (pre-ejection shortening) and rightward septal motion (early systolic lengthening) within the isovolumic contractile period (Fig. 2). Patients with LBBB were allocated into two subgroups: patients with SF (LBBB-SF) and patients without SF (LBBB-NSF). The extent and magnitude of SF was assessed. The extent of SF was scored as the number of the involved septal myocardial segments. The magnitude of SF was defined as the maximal early negative peak strain in the involved septal myocardial segments.

### Intra-observer and inter-observer variability

Intra-observer and inter-observer variability for echocardiography assessment of SF and RV LS were examined in 20 randomly selected patients from the LBBB-SF group. The same observer who was blinded to the initial measurements repeated the measurements after more than four weeks had elapsed, to assess intra-observer variability. In addition, a second independent observer repeated the measurements twice to assess inter-observer variability. A third observer resolved any disagreements in SF assessment.

### Statistical Analysis

Statistical analysis was performed using the SPSS 17.0 software package. Continuous data were expressed as the mean ± standard deviation, and frequency and percentage were set as categorical variables. The Student’s t-test was used to compare normally distributed continuous variables. Categorical variables were compared using the chi-squared test. Correlations were sought using Spearman and Pearson correlation analyses where appropriate. The relationships between continuous variables and other variables were analyzed using simple linear regression analysis. The Bland-Altman analysis was used to estimate intra- and inter-observer variability. For all parameters, a value of p < 0.05 (two-tailed) was considered statistically significant.

## Results

Of 83 potential study individuals, one (1.2%) patient was excluded due to poor ECG quality, which may have been a result of an unstable ECG signal. During the analysis of RV strain by 2D STE, five (6.0%) patients with LBBB and three (3.6%) controls were excluded because of poor echocardiographic images and inadequate tracking quality of more than one segment in the RV-focused apical four-chamber view. The data of the remaining 74 subjects (44 LBBB patients with preserved LVEF and 30 controls) were used for statistical analysis.

### Left bundle-branch block and septal flash

Baseline characteristics of the study population are shown in Table 1. There were no differences in baseline characteristics between the groups. Of the 44 LBBB patients with preserved LVEF, 24 (54.5%) had SF. Three-, four-, five-, and six-segment involvement of SF was found in 25.0%, 4.2%, 16.7%, and 54.1% of patients, respectively. There were no patients with one- or two-segment involvement of SF. The mean value of the magnitude of SF was −5.87 ± 2.69%.

**Table 1:** Baseline characteristics of the study population.

Variable	LBBB (n = 44)	Controls (n = 30)	*P*-value
Age (years)	58.4 ± 10.9	57.9 ± 8.0	0.72
Male sex (%)	17 (39%)	14 (47%)	0.49
Body surface area (m^2^)	1.72 ± 0.18	1.69 ± 0.19	0.54
Heart rate (beat/min)	76.0 ± 11.5	73.8 ± 10.4	0.31
Systolic blood pressure (mm Hg)	126.4 ± 10.6	124.0 ± 12.8	0.40
Diastolic blood pressure (mm Hg)	77.4 ± 7.5	78.8 ± 9.3	0.32
Fasting blood glucose (mmol/L)	5.49 ± 1.11	5.38 ± 0.82	0.58
LDL cholesterol (mmol/L)	2.79 ± 0.72	2.66 ± 0.57	0.29
Triglycerides (mmol/L)	1.37 ± 0.73	1.39 ± 0.54	0.83
HDL cholesterol (mmol/L)	1.15 ± 0.23	1.17 ± 0.31	0.83
Total cholesterol (mmol/L)	4.39 ± 0.83	4.26 ± 0.76	0.37
LV ejection fraction (%)	60.36 ± 5.43	63.60 ± 4.72	0.01
LV global LS (%)	−18.46 ± 2.93	−20.83 ± 1.67	<0.001
QRS width (ms)	156.08 ± 11.14	110.80 ± 12.60	<0.001
Septal flash (%)	24 (54.5%)	0 (0%)	<0.001
Previous history of hypertension	4 (9.1%)		
Previous history of hyperlipemia	5 (11.4%)		
Medications:			
ACEI/ARB (%)	3 (6.8%)		
Beta-blockers (%)	1 (2.3%)		
Calcium channel blocker (%)	2 (4.5%)		
Statin (%)	3 (6.8%)		

Values shown are Mean ± SD or percentage. LV, left ventricle; LDL, low-density lipoprotein; HDL, high-density lipoprotein; LS, peak systolic longitudinal strain; ACEI, angiotensin-converting enzyme inhibitor; ARB, angiotensin II receptor blocker.

The QRS duration among patients with or without LBBB differed significantly. LBBB patients with preserved LVEF had a wider QRS duration (p < 0.001). Further, the QRS duration of LBBB-SF patients was wider than LBBB-NSF patients (158.66 ± 11.04 ms vs. 152.82 ± 10.66 ms, p = 0.03).

### Septal flash and LV systolic function

LBBB patients had a lower LVEF, although LVEFs of both groups were in the normal range (Table 1). LVEF of LBBB-SF patients was lower than LBBB-NSF patients (58.65 ± 4.64% vs. 62.41 ± 5.70%, p = 0.02). LVEF negatively correlated with septal myocardial segments of SF (r = −0.38, p = 0.01), but did not correlate with the magnitude of SF (r = 0.14, p = 0.52). LBBB patients had lower LV global LS (Table 1). LV global LS of LBBB-SF patients was lower than LBBB-NSF patients (−17.62 ± 3.22% vs. −19.47 ± 2.22%, p = 0.01). LV global LS negatively correlated with septal myocardial segments of SF (r = −0.41, p = 0.006), but did not correlate with the magnitude of SF (r = 0.19, p = 0.37).

### Septal flash and RV contractileperformance

Values for RV chamber dimensions and contractile performance of the study population are presented in Table 2. RV LS was significantly lower in LBBB patients with preserved LVEF than in control subjects. Conversely, RIMP was found to be higher in LBBB patients with preserved LVEF. However, there were no differences in tricuspid S’ and TAPSE between groups.

**Table 2:** Effect of SF on RV contractile performance in LBBB patients with preserved LVEF.

Variable	LBBB (n = 44)	LBBB-SF (n = 24)	LBBB-NSF (n = 20)	Controls (n = 30)	*P*-value, LBBB vs.Controls	*P*-value, LBBB-SF vs. LBBB-NSF vs.Controls
RVD1 (mm)	32.88 ± 3.12	32.83 ± 2.52	32.94 ± 3.80	31.62 ± 2.59	0.56	0.51
RVD2 (mm)	26.50 ± 3.25	26.44 ± 3.04	26.57 ± 3.57	25.99 ± 3.04	0.32	0.30
TAPSE (mm)	21.56 ± 2.93	21.48 ± 2.67	21.85 ± 2.74	22.56 ± 2.41	0.13	0.10
Tricuspid S′ (cm/s)	11.48 ± 2.36	11.17 ± 2.39	11.85 ± 2.32	11.70 ± 1.60	0.65	0.51
RVFAC (%)	55.08 ± 4.55	54.68 ± 3.90	55.97 ± 3.51	56.47 ± 4.94	0.22	0.20
RIMP	0.64 ± 0.21^*^	0.69 ± 0.20^*§^	0.58 ± 0.20^*^	0.45 ± 0.11	<0.001	<0.001
RV LS (%)	−29.54 ± 6.04^*^	−28.03 ± 6.66^*§^	−31.43 ± 4.65	−33.59 ± 4.30	0.002	0.001

Values shown are Mean ± SD. RV, right ventricle; RVD1, RV basal cavity diameter;RVD2, RV mid cavity diameter; TAPSE, tricuspid annular plane systolic excursion; RVFAC, RV fractional area change; RIMP, RV index of myocardial performance; LS, peak systolic longitudinal strain.

^*^ *P* < 0.05 versus control group;

^§^ *P* < 0.05 versus LBBB-NSF group.

**Figure 3:**
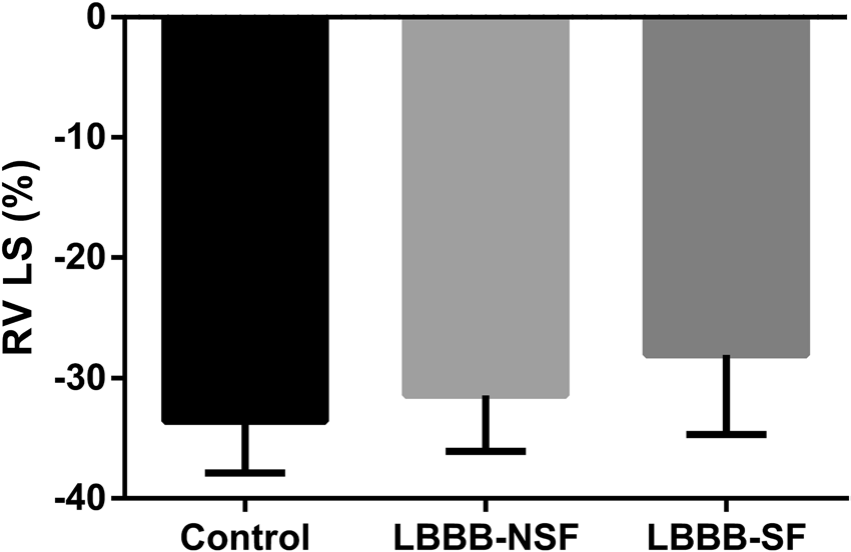
Effect of SF on RV LS. Note that RV LS of the LBBB-SF patients was significantly lower than the LBBB-NSF patients and controls. SF, septal flash; RV, right ventricle; LS, longitudinal strain.

The effect of SF on RV contractile performance in LBBB patients with preserved LVEF was further analyzed. RV LS of the LBBB-SF patients was significantly lower than controls and the LBBB-NSF patients (p = 0.04, Fig. 3). The presence of SF was the main determinant of RV contractile performance in LBBB patients with preserved LVEF (Table 3).

**Table 3:** Determinants of RV LS in LBBB patients with preserved LVEF.

	*P*-value	β	95% CI
Age	0.09	−0.15	−0.32–0.03
Gender	0.79	−0.51	−4.42–3.39
QRS duration	0.87	0.02	−0.16–0.19
LVEF	0.70	0.07	−0.31–0.45
Presence of septal flash	0.04	−4.04	−8.10–0.02

RV, right ventricle; LS, peak systolic longitudinal strain; LVEF, left ventricular ejection fraction.

RV LS correlated negatively with involved septal myocardial segments of SF (r = −0.36, p = 0.02), but did not correlate with the magnitude of SF (r = 0.14, p = 0.51).

### Reproducibility

The intra-observer and inter-observer concordances on identifying SF by 2D STE were 20/20 (100%) and 19/20 (95%). Mean differences of intra-observer and inter-observer variability of RV LS were 0.48% (95% CI: −1.04 to 0.72%) and 0.82% (95% CI: −1.66 to 1.04%).

## Discussion

The findings of the present study can be summarized as follows: (1) SF was present in 54.5% of LBBB patients with preserved LVEF; (2) RV contractile performance deteriorated in LBBB patients with preserved LVEF; (3) SF may further affect RV contractile performance of LBBB patients with preserved LVEF, and may be the main determinant; (4) the degree of the impairment of RV contractile performance in LBBB patients with preserved LVEF correlates with the extent (but not the magnitude) of SF.

The mechanisms of septal flash (abnormal pre-ejection leftward motion and following rightward motion) during LBBB were expounded by Gjesdal *et al*.^5^ using an experimental model. They attributed the leftward motion to septal active contraction, through analysis of pressure-segment length loops, and showed that delayed LV lateral wall contracting, septal flattening, and RV volume reduction may be due to the paradoxical rightward motion of the septum. However, in patients with LBBB diagnosed by current, new, and rigorous ECG criteria, SF does not always appear. In this study, we investigated the prevalence of SF (54.5%) in LBBB patients with preserved LVEF, the results of which were different from a study by Corteville *et al*. (45.2%)^12^. In their study, 125 LBBB patients with a wider range of LVEF (37%–61%) were selected. Differences in the restriction point of LVEF in the study population, and the sample size, may explain the difference in SF prevalence. In our study, we selected patients with normal ranging LVEF to avoid the effect of LV pump function on RV contractile performance.

In addition to the presence of SF, we counted the involved myocardial segments of SF and measured the magnitude of SF in each patient in the LBBB-SF group. The presence, extent, and magnitude of SF were varied in LBBB patients. All LBBBs were not absolutelyequal. This heterogeneity may relate to the variable anatomy of the left bundle, the different sites of LV breakthrough, and the different levels and/or extent of conduction block in the left bundle^1, 6^. Moreover, septal infraction and other factors that can affect the transseptal pressure gradient may also affect the presence and magnitude of SF^5, 12, 13^.

A previous study by Corteville *et al*.^12^ revealed a significant correlation between the presence of SF and longer QRS duration. In line with their findings, we also found that the QRS duration of LBBB-SF patients was wider than LBBB-NSF patients and controls. In spite of this, surface ECG recordings may not be adequate to precisely identify the presence, extent, and magnitude of SF, which highlights the advantages of echocardiography in identifying further subtypes of LBBBs.

Other studies have been reported regarding the effect of LBBB on LV function. Electric activation asynchrony as a result of LBBB may lead to abnormal inter-ventricular, intra-ventricular, and atrioventricular coupling, which impairs the ability of the LV to fill or eject blood. In addition to its direct effect on LV mechanical function, asynchronous electric activation may affect coronary flow throughout the coronary vasculature from the epicardial arteries to the microvascular bed. This can lead to subsequent changes of myocardial perfusion, oxygen demand, glucose metabolism, and have an ultimately deleterious effect on LV function during LBBB^14–17^. Moreover, asynchronous electric activation may lead to LV remodeling caused by regional differences in workload, including asymmetric myocardial hypertrophy and LV dilatation, which may aggravate preexisting LV pump performance^18^. When SF occurs, septal active contraction, followed by lateral wall passive stretch and delayed lateral wall shortening, followed by septal lengthening, has a further negative effect on LV stroke volume, which is similar to the effect of an aneurysm during LBBB^5^. In line with these findings, we also found that the LVEF of LBBB patients was lower than controls, and that LBBB-SF patients had a lower LVEF than LBBB-NSF. Additionally, we found the LV global LS of LBBB-SF patients was lower than LBBB-NSF and controls.

Compared to LV, studies about RV function in patients with LBBB in the published literature are limited. A previous study by Kuhn *et al*.^19, 20^ suggested that LBBB patients with normal LV dimensions and EF at rest may present with an abnormal increase in mean pulmonary artery pressure during exercise. Moreover, they performed myocardial biopsy from the RV septum and found abnormal ultrastructural findings in myocardial cells, such as degeneration, interstitial fibrosis, mitochondrial and myofibrillar changes, among others, in LBBB patients with normal LV. However, thus far, no study has provided data about RV myocardial systolic performance using a reliable method in LBBB patients with preserved LVEF. 2D STE is a novel technique enabling more reliable assessment of RV myocardial performance by obtaining myocardial strain, which is angle independent and less confounded by overall heart motion. In this study, we measured RV LS using 2D STE and found that RV contractile performance decreased in LBBB patients with preserved LVEF. The reasons for these changes are not well understood. We speculated preliminarily that, besides LV, electric activation asynchrony caused by LBBB may also lead to RV mechanical asynchrony, an abnormal perfusion of the right coronary artery, and myocardial remodeling of the RV. These remain to be explored in a series of large future studies.

In the current study, we found SF to be the main determinant of RV contractile performance, and the more involved the segments of SF were in the septal myocardium, the worse the RV contractile performance was in LBBB patients with preserved LVEF. However, the magnitude of SF did not correlate with RV contractile performance. These findings may relate to the different levels of conduction block within the left bundle conduction system. It has been revealed that patients with a large SF may have a proximal block in the left bundle, longer transseptal conduction time, and a greater degree of inter-ventricular mechanical delay^6, 21^. However, patients with no SF had a shorter transseptal conduction time, probably because of activation within the proximal left bundle; this correlated more with intra-ventricular mechanical delay. It was well known that inter-ventricular mechanical asynchrony may affect both LV and RV function; but intra-ventricular mechanical asynchrony may not have a direct effect on RV function.

It appears that LBBB occurs in several cardiac conditions, such as dilative cardiomyopathy or myocardial infarction, and may aggravate preexisting conditions. However, in some cases, LBBB may be isolated without any other abnormal findings^18^. Previous researchers found normal LV function, abnormal ultrastructural changes of myocardial cells from the RV septum at rest, and slight changes during exercise in patients with isolated LBBB, and they suggested that LBBB may be the early stage of a dilative cardiomyopathy^19, 22^. In the current study, we revealed abnormal RV contractile performance in LBBB patients with normal LVEF at rest. This emphasizes the importance of paying attention to these patients, especially those with a large extent of SF, and monitoring RV function by measuring RV LS, which may provide a theoretical basis for timely clinical treatment and management before the patient develops dilative cardiomyopathy or other worse conditions.

### Study Limitations

A major limitation of this study was that the manufacturers had not yet developed dedicated software for RV analysis. Software for measuring RV LS has been designed for LV measurements. However, recent studies have shown that the evaluation of RV deformation using 2D STE by the same software is feasible and reproducible^10, 23, 24^.

Another limitation was that the measurement of RV LS was from a single image plane (RV-focused apical four-chamber view) because of the complex RV geometry and limited acoustic window. We would have preferred to obtain LS from the complete RV cavity.

Moreover, we found that the extent of SF correlated with RV contractile performance. However, we did not define high-risk clinical criteria for involved myocardial segments of SF, which could have identified patients whose RV performance required special attention. In future studies, we will use larger sample sizes and make follow-up observations of the patients.

## Conclusions

RV contractile performance decreased in LBBB patients with preserved LVEF. SF, particularly the extent of the phenomenon, may further affect RV contractile performance in these patients.
